# Thermal Weathering of 3D-Printed Lunar Regolith Simulant Composites

**DOI:** 10.1021/acsaenm.4c00158

**Published:** 2024-07-27

**Authors:** Alexandra Marnot, Jami Milliken, Jaehyun Cho, Zihao Lin, Chingping Wong, Jennifer M. Jones, Curtis Hill, Blair Brettmann

**Affiliations:** † School of Chemical and Biomolecular Engineering, Georgia Tech, Atlanta, Georgia 30332, United States; ‡ Daniel Guggenheim School of Aerospace Engineering, Georgia Tech, Atlanta, Georgia 30332, United States; § School of Materials Science and Engineering, Georgia Tech, Atlanta, Georgia 30332, United States; ∥ NASA Marshall Space Flight Center, Huntsville, Alabama 35806, United States; ⊥ NASA Marshall Space Flight Center, Jacobs Space Exploration Group, Huntsville, Alabama 35806, United States

**Keywords:** regolith simulant, composites, lunar weathering, 3D printing, ISRU

## Abstract

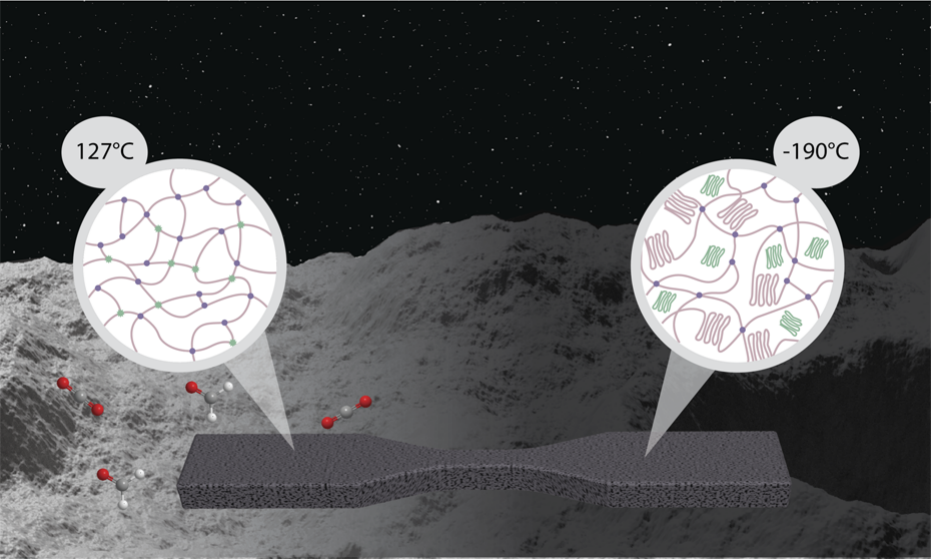

The production of lunar regolith composites is a promising venture, especially when enabled by extrusion-based additive manufacturing techniques such as direct ink write. However, both three-dimensional (3D) printing production and usage of polymer composites containing regolish on the lunar surface are challenges due to harsh environmental conditions such as severe thermal cycling. While thermal degradation in polymer composites under thermal cycling has been studied, there is limited understanding of how polymer properties impact the mechanical performance of lunar regolith composites when both printing and usage are carried out under extreme thermal conditions. Here, we aim to bridge that gap through the creation of composites containing a lunar Highlands regolith simulant suspended in an ultraviolet (UV) curable binder, which were printed at −30 °C and thermally cycled between weekly lunar day (127 °C) and weekly night (−190 °C) temperatures. We validate that thermal stresses cause both physical and chemical degradation since the regolith simulant composites become stiffer, more porous, and show yellowing after exposure to thermal cycling. Moreover, we indicate that chemical degradation mechanisms seem to compete with residual polymerization in certain formulations. We attribute this phenomenon to partial crystallization of monomer species during printing at −30 °C, resulting in low vinyl bond conversion during initial curing. The results presented here shed light on the intricate interplay between thermal stresses, uncured polymer properties, and degradation mechanisms, which can help guide future use cases of regolith composites for lunar infrastructure needs.

## 1 Introduction

In situ resource utilization (ISRU) is a pillar for economically feasible infrastructure development on the surface of other planetary bodies. Currently, with a focus on the lunar surface due to the Artemis program, most ISRU applications are centered around the use of a lunar regolith. Ranging from metal extraction for electronics fabrication to oxygen extraction for breathable air, to use of the regolith as-is for cement substitution,^1,2^ ISRU efforts are both varied in scope and in technique. Of particular interest is combining ISRU of regolith particles with extrusion-based additive manufacturing (AM) methods to implement rapid, customizable, and on-demand infrastructure.^3^ For instance, a collaboration between NASA, ICON, and academic institutions demonstrated the use of extrusion three-dimensional (3D) printing with lunar regolith simulants to create the Lunar Plume Alleviation Device (PAD).^4^ Another example, from the European Space Agency’s Spaceship European Astronaut Centre (EAC) program, demonstrated custom filaments incorporating regolith simulants for fused filament fabrication.^5^ But as these new formulations are being developed, so too is the need to account for their processability in the unique environmental conditions of space. For the lunar surface, this includes vacuum, microgravity, thermal extremes, solar and cosmic radiation as well as dust and charging.^6−8^ Therefore, ISRU formulations for AM need to be developed with components that have the least complexity possible and result in few challenges to processing in harsh environmental conditions. This approach will limit the need for extra additives from Earth or significant equipment modifications in already isolated environments.

Proper material selection for extrusion-based printing inks containing regolith to be successful feedstocks for manufacturing in harsh environments is only a part of the challenge. Regolith parts produced by extrusion AM must also be weatherable to thermal extremes, vacuum, radiation, etc., and information tying the composition of the parts to weatherability is essential to enable rapid and enduring design. However, new inks designed for extrusion in harsh environments have not yet been evaluated for their stability in the unique and harsh lunar environment. Most formulation designs for extrusion-based AM containing regolith simulants are composite materials, where the regolith is the solid phase, and a binder such as a prepolymer or monomer is added to suspend the solids and help with flow during extrusion. Polymer binders are particularly attractive with regard to ISRU since they can be derived from biological or waste byproducts already brought to the lunar surface.^9−12^ In some cases, the liquid polymer present in the regolith composite is burnt out after processing, such as when requiring highly conductive prints in electronics fabrication,^13^ but there are cases where it is desirable to maintain the composite composition after printing, such as when requiring the polymer for radiation shielding application.^14,15^ In such cases where the final printed part does not undergo binder burnout, repeated exposures to extreme temperatures, radiation, and vacuum are incredibly important to assess because polymers tend to be more sensitive to degradation by radiation, temperature, etc. than inorganic material.^16,17^

In addition to polymer binder degradation, the nature of the inhomogeneous properties in a composite can lead to stresses that worsen during aging processes. For example, under exposure to thermal extremes, composite materials that have different coefficient of thermal expansions will bear thermal stresses differently than pure materials and this can result in premature and uncontrolled failure.^18^ Formulations that contain regolith may require even further thermal characterization, as current simulants are known to be composed of at least 10 metal oxides, all of which will have varying responses to thermal stress.^19,20^ Understanding how composites behave under weathering is therefore crucial to predicting their mechanical properties over their service life.

On the Moon, temperatures can vary by as much as 290 °C between lunar daytime and night, due to the lack of an atmosphere.^21^ At the rim of Shackleton Crater, one of the targeted landing sites of the Artemis program at the lunar South Pole, daytime temperatures can reach as high as 57 °C, while nighttime temperatures (nighttime lasting 6−9 days) can fall to a low −194 °C.^22,23^ At the equator, these temperatures can reach as high as 127 °C.^21^ Therefore, parts made from regolith–polymer formulations must be evaluated for repeated usage under these thermal conditions. Fortunately, the performance of composites exposed to thermal cycling has been extensively studied.^24−29^ During long heating periods, polymer binders are susceptible to degradation, particularly in the presence of oxygen. Mechanisms such as chain scission, and subsequent cross-linking have been demonstrated for fiber-reinforced composites with thermoset epoxy-based binders.^30^ Continued polymerization was observed for ultraviolet (UV) curable resins used in glass composites.^31^ For thermoplastic polymer binders, the ratio of crystalline to amorphous regions is a driving factor for resistance to thermal cycling, as was shown for a polyethylene/Mars regolith material.^14^ On the other hand, during cooling cycles below 0 °C, the main concern for composite materials is the contraction of the binder leading to delamination between layers and deadhesion between the binder and the particles.^25,30^ This is a phenomenon that has been observed for carbon fiber-reinforced composites, where it results in the formation of microcracks that weaken the structural integrity of the materials and decreases the strength of parts.^30,32^ These microcracks can also accelerate oxidative degradation of the binder, showing how the combined effect of cold and hot cycles can severely deteriorate composite parts.^29^

The examples listed here highlight the importance of evaluating the thermal aging characteristics of any binder selected for lunar regolith formulations. However, to the best of our knowledge, no assessment of the effect of thermal weathering has been conducted on lunar regolith simulant composites not produced in ambient conditions. While environment-controlled habitats will likely house several manufacturing processes, enabling production in extreme thermal conditions can facilitate exploration on other planets, such as Mars. Still, there is a knowledge gap regarding the performance of regolith composite parts produced at extreme temperatures and subsequently utilized in harsh thermal cycles. In our past work, we demonstrated that printing of high solid inks with UV curable binders could be achieved at a replicated lunar temperature of −30 °C, and that printing at subzero temperatures significantly affected the way different ratios of small and large monomers cross-link, as compared to ambient printing.^33^

Here, we seek to assess the weatherability of parts produced via this process when exposed to replicated maximum lunar day and minimum night conditions (127 °C during the daytime and −190 °C during nighttime). We consider similar binder materials as previously used, consisting of the larger prepolymer poly(ethylene glycol) diacrylate (PEGDA) and the smaller monomers hexyl acrylate (HA) and 1,6 hexanediol diacrylate (HDDA). Similar molecules of the monofunctional HA monomer and difunctional HDDA monomer allow for a comparison of changing cross-linking densities while keeping the molecular structure fairly constant. In addition, we prioritize high cross-linking densities with difunctional monomers (PEGDA and HDDA) as they produce prints with higher strength and toughness, which are composite properties previously shown to exhibit increased resistance to thermal cycling.^34,35^ Our binder formulations are varied to contain either 1:1 or 2:1 v/v PEGDA/monomer, with either HDDA or HA as the monomer. Our solids formulation is a mixture of glass microspheres used in our prior works and lunar Highlands regolith simulant. The overall printing inks contain 60 vol % solids and are printed at −30 °C. We aim to evaluate how minor changes in the binder formulation, such as monomer ratio and functionality, influence the weathering capacity of lunar regolith inks by comparing the tensile properties and the porosity of prints subjected to thermal cycling with those that were not exposed to such cycling.

Although the regolith simulant content in this work remains on the lower end (38 wt %) than what would be desired for the full scope of ISRU, we provide here new insights on material characterization for both processing and usage in extreme environments. We expect that as lunar infrastructure develops, the ISRU content in these inks could be improved including with the use of biobased binders such as acrylated vegetable oils^36,37^ grown in farming modules. Likewise, we anticipate that with the efforts towards metal extraction from the regolith, possibly from the regolith particles that were sieved out, SiO_2_ particles could be produced for incorporation into these inks. In all, we expect that the use of regolith as demonstrated with the simulant in this work will be paired with other ISRU efforts, such that over time the reliance on earth-based materials will decrease. By first demonstrating the implications of sample binder formulation on the service life of parts printed in extreme cold and subjected to the thermal stresses of lunar day and night, we aim to offer additional methods of processing and utilizing regolith while minimizing the need for environmental control.

## 2 Materials and Methods

### 2.1 Particle-Reinforced Composite Formulation

Inks were composed of a mixture of lunar Highlands simulant (LHS-1, Exolith Lab) and glass microspheres (A-1820, Potters Industries) to optimize the penetration of light through tall layer heights and to ensure curing.^38^ A bimodal particle size distribution was used to optimize particle packing despite the shard-like shape of the regolith simulant, which allowed a solid loading of 60 vol % when using a 65:35 ratio of glass microspheres to LHS-1. Glass microspheres, while not native to the lunar surface, can be replaced in the future with regolith glass or SiO_2_ byproducts of regolith metal extraction. The spherical shape of the particles allows particles to pack more efficiently and reduces friction between particles, but this particle morphology will likely not be achievable on the moon without complex machinery. The rougher and irregular particle shapes, which would most likely be obtained instead from regolith glass or SiO_2_ byproducts, would require further optimization of the size distribution of the particles in order to maintain both high solid loadings and ink flowability. The LHS-1 particles were sieved using a #300 stainless steel sieve to bring the particle size to under 53 μm and produce the smaller component of the bimodal particle size distribution. We expect that sieving will still be needed on the lunar surface to maintain the bimodal ratio established here and so that the solid loading in the inks can remain high. Particle size analysis of the sieved LHS-1 particles conducted via a Malvern Hydro 2000SM particle size analyzer measured *d*_10_, *d*_50_, and *d*_90_ as 3, 22, and 50 μm, respectively. For the glass microspheres, *d*_10_, *d*_50_, and *d*_90_ were 231, 271, and 317 μm, respectively.

**Figure 1:**
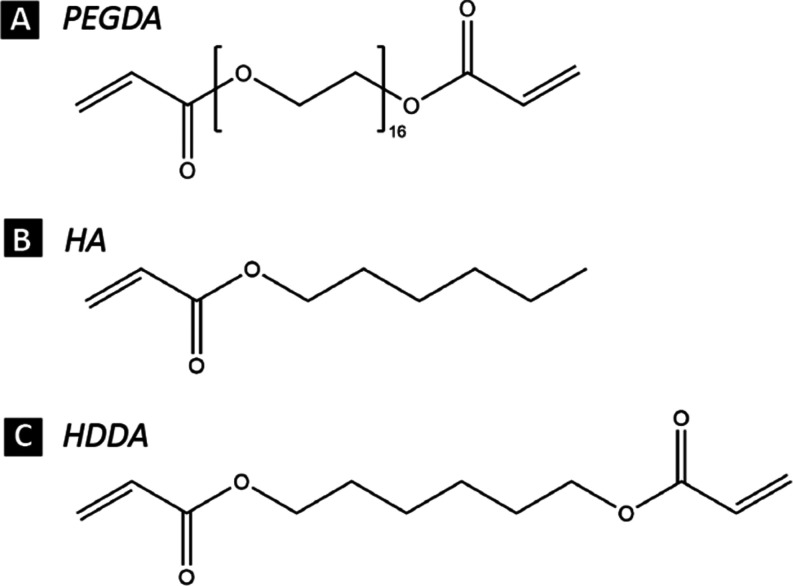
Molecular structures of UV cure monomers. (A) poly(ethylene glycol) diacrylate, PEGDA, (B) hexyl acrylate, HA, and (C) 1,6 hexanediol diacrylate, HDDA.

The polymer binders were composed of a mixture of monomers used in our prior work,^33^ which included poly(ethylene glycol) diacrylate (PEGDA, Sigma-Aldrich), a larger prepolymer, and either hexyl acrylate (HA, Sigma-Aldrich) or 1,6 hexanediol diacrylate (HDDA, Sigma-Aldrich) as the second component (Figure 1). The PEGDA-to-monomer ratios used were 1:1 and 2:1, which provided an additional level of control over the cross-linking densities. The photoinitiator phenylbis (2,4,6-trimethylbenzoyl)-phosphine oxide (BAPO, Sigma-Aldrich) was added to the particle and binder mixture as the last component at a concentration of 0.5 wt % on the binder weight.

Corresponding weights of the particles, binder, and photoinitiator are shown for each formulation in Table 1 for 12 mL batches. The completed mixtures were then mixed in a Flacktek 400.2VAC-L dual-axis centrifugal (DAC) mixer at 1000 rpm for 1 min, after which the inks were kept stationary at 0 rpm for 45 s, and a final mixing step at 1500 rpm for 45 s was then applied.

**Table 1:** Ink Composition for Each of the Formulations^a^

formulation	small particles (g)	large particles (g)	PEGDA (mL)	HA (mL)	HDDA (mL)	BAPO (g)
1:1 PEGDA/HA	7.41	11.7	2.4	2.4		0.0237
2:1 PEGDA/HA	7.41	11.7	3.2	1.6		0.0247
1:1 PEGDA/HDDA	7.41	11.7	2.4		2.4	0.0247
2:1 PEGDA/HDDA	7.41	11.7	3.2		1.6	0.0253

a Small particles are the lunar regolith simulant LHS-1, while large particles are glass microspheres A-1820. PEGDA is the prepolymer poly(ethylene glycol) diacrylate, HA is the monomer hexyl acrylate, HDDA is the monomer 1,6 hexanediol diacrylate, and BAPO is the photoinitiator phenylbis (2,4,6-trimethylbenzoyl)-phosphine oxide.

### 2.2 −30 °C UV-Direct Ink Write

Printing of the ASTM D638 Type V dogbones was performed on a Hyrel Engine Standard Resolution (ESR) printer outfitted with a Volcano 25 print head, a 14G tapered UV-blocking nozzle, and a 365 nm UV array positioned around the nozzle. The printer itself was housed within an enclosed chamber through which cold and warm N_2_ gas could be cycled to cool or warm the chamber. Thermocouples located within the chamber monitored the temperature, which was kept at a constant −30 °C by the flow of cold N_2_ gas through a solenoid valve activated by a custom LabView program. Inks were mixed in ambient conditions, as described above, and were immediately loaded into the Volcano 25 syringe barrel. No degassing step was necessary as trapped air could escape with the syringe loaded in a bottom-up approach. The chamber was closed after positioning and calibration of the print head and purged of air prior to cooling (purge time was approximately 20 min). Printing of ASTM D638 type V dogbones was performed once the chamber reached −30 °C, approximately 1.5 h after purging the air. The print speed was set to 10 mm/s with an extrusion rate of 260 pulses/μL and a layer height of 1 mm. The syringe barrel and nozzle were kept heated between 20−40 °C as described in our past work utilizing this system.^33^ Following the completion of each print, the print head was manually positioned over the center of the sample, and a UV pen emitting 17 W/m^2^ was activated to postcure the print in situ at −30 °C. Minor movements along the *x*-axis of the printer allowed the entirety of the print to be exposed to the higher-intensity UV light for a duration of 1.5 min. After the postcure step, the chamber was heated back to ambient conditions and opened, and the prints were retrieved. Three printed dogbones were printed in each round of cooling of the chamber, and a total of nine dogbones were printed for each formulation.

### 2.3 Thermal Cycling

**Figure 2:**
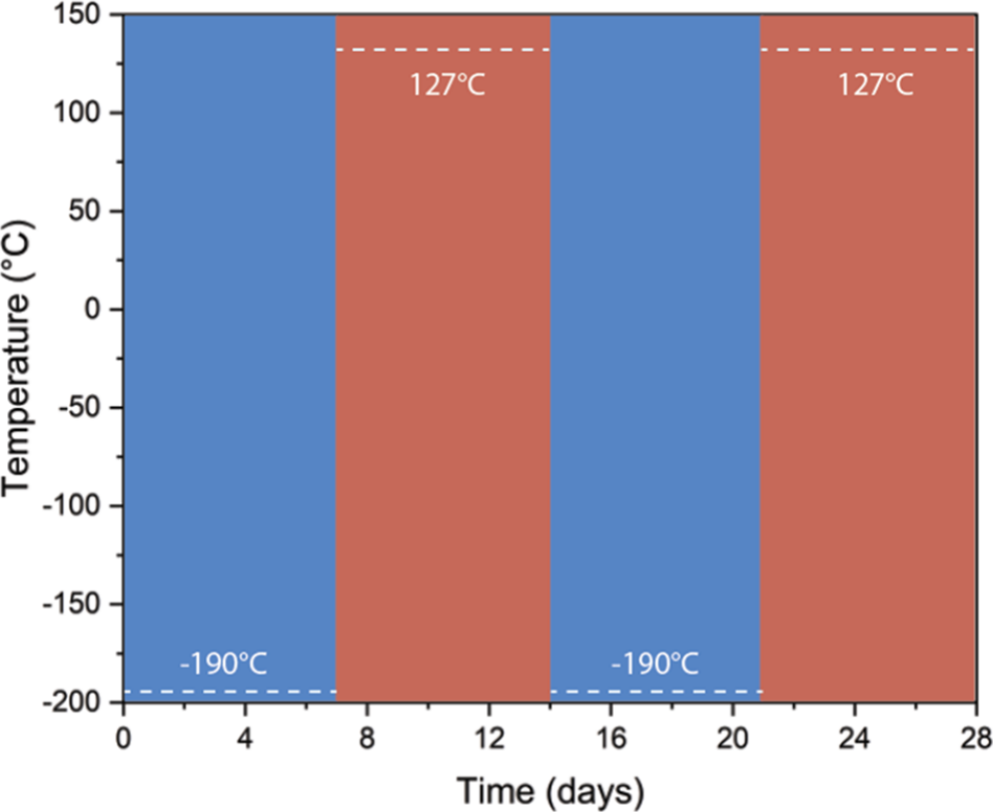
Thermal cycling experiment time intervals at the replicated lunar night (blue) and day (red) temperatures.

Out of the nine dogbones printed for each formulation, five were randomly selected to undergo thermal cycling, while the remaining four were kept as the control group. The thermal cycled (TC) samples were exposed to simulated lunar day/night temperatures as shown in Figure 2. The cold cycles were performed by placing the samples in holders in a liquid nitrogen dewar and filling the bottom 1/3 of the dewar with liquid nitrogen. Thermocouples at various points in the dewar and along the sample holders monitored the temperature and indicated the need to refill the dewar. The heating cycles were carried out immediately after the samples were removed from the dewar and placed in a convection oven with an air atmosphere. For the duration of the thermal cycling treatment described in Figure 2, the control samples were stored at room-temperature conditions (23 ± 2 °C) in airtight containers and away from light.

### 2.4 Tensile Testing

Assessment of the tensile properties of the dogbones was carried out within 1 week of the completion of the thermal cycling treatment (4+ weeks after printing). A DHR 3 (TA Instruments) was equipped with a rectangular tension accessory, and the dogbones were tested according to the ASTM D638 standard. The pull rate was set to 5 mm/min, and all nine samples (five TC and four control) for each formulation were tested.

### 2.5 Microcomputed Tomography

Within 1 week of the completion of the thermal cycling conditioning, the samples were imaged via microCT. Imaging of the entire dogbones was performed on a North Star Imaging X5000 scanner with voltage and current settings of 80 kV and 300 μA, respectively, a 4× frame averaging, and 1394 projections. A copper filter was also used. Reconstruction was achieved in the native software, and visualization was realized in the VGStudio software. Two dogbones of each formulation and each thermal treatment were imaged and are shown in SI Figure 1. These scans offered visualization of the whole dog-bone prints at the cost of a higher voxel resolution of 40 μm. Thus, additional scans were carried out on smaller sections of the dogbones on a Scanco μ50 microCT to achieve a higher resolution of 4 μm/voxel and visualize the smaller regolith simulant particles. For this instrument, the voltage and current settings were set to 55 kV and 72 μA, a 0.5 mm aluminum filter was utilized, and an integration time of 1000 ms/slice for 1000 slices. Reconstruction was performed in the Dragonfly software, along with the porosity analysis that used Otsu’s method for thresholding voids and particles/binder. Otsu’s method relies on identifying a gray value threshold (between 0 and 255) that sufficiently differentiates a grayscale image’s “foreground” and “background”. This requires the scanned image’s histogram, and the thresholding software will numerically detect this threshold gray value by minimizing the weighted sum of the variance in both the “foreground” and “background” classes, while maximizing the variance across the two classes.^39,40^ In the analysis of porosity of the printed samples, the foreground was denoted as the pixels forming the nonpore regions (binder and particles) and the background was the pixels forming the void region. This analysis was performed on three scans for each formulation and thermal treatment.

### 2.6 FTIR

Samples for Fourier transform infrared (FTIR) spectroscopy were prepared by crushing the printed parts and by mixing the crushed pieces to allow for a sampling representative of the whole print volume. A Spectrum Two instrument from PerkinElmer (UATR Two) was utilized to carry out attenuated total reflectance Fourier transform infrared-spectroscopy (ATR-FTIR). Scans were run from 4000 to 500 cm^−1^ at a resolution of 4 cm^−1^ with an average of 32 scans for each formulation for both the control and TC samples. A baseline correction was applied with a background scan of the ambient atmosphere prior to the measurements. Three samples were run for each formulation in both the control and TC group.

### 2.7 Photo-DSC

**Figure 3:**
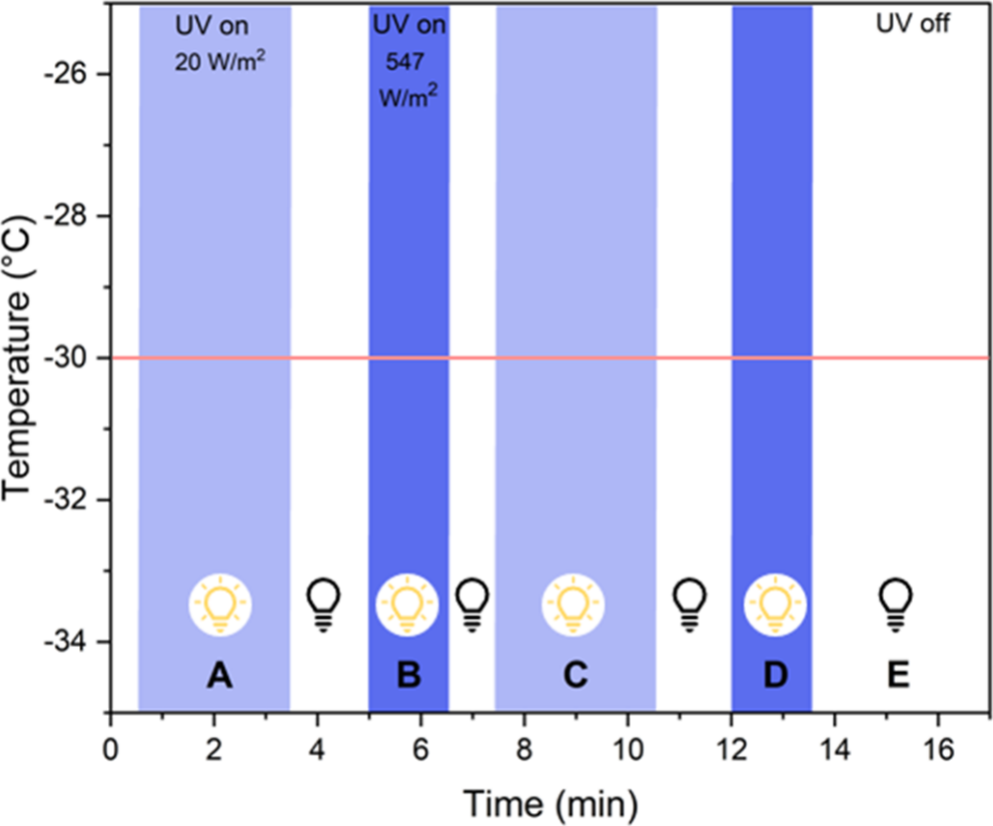
Intervals in the photo-DSC experiment. (A, C) Low UV intensity intervals shown in light blue correspond with UV exposure during extrusion, while (B, D) darker blue intervals show the higher UV intensity triggered during the postcure step at the end of printing. The section after the UV lamp is turned off is labeled as panel (E). The intervals are repeated to deconvolute heating effects from the mercury lamp.

Samples were loaded into 40 μL aluminum pans and tested in a Mettler Toledo 3+ differential scanning calorimeter (DSC) fitted with an Omnicure 2000 UV lamp with a 365 nm filter. The furnace temperature was set to −30 °C, the target printing temperature. The relative intensity of the UV lamp was set to 20 W/m^2^ to mimic the UV dosage received during the printing and postcuring processes. Curing was performed in the intervals shown in Figure 3, which consist of an initial UV dose with 3 min of exposure (A), followed by a period of a higher UV dose (547 W/m^2^) for 1.5 min (B). These steps were repeated for another cycle (C and D) in order to deconvolute the heat recorded by the polymerization reaction from heating due to the mercury lamp. Finally, a baseline step with no UV exposure and no curing reaction was recorded (E).

Degree of cure was computed from the measured enthalpy of cure at specific time intervals and compared with the full curing enthalpy of the binder itself. The calculation follows eq 1.^41−43^ For the full curing enthalpy of the binder, Δ*H*_binder_, preliminary tests were run on the binders mixed with the same photoinitiator amount as described for the regolith simulant formulations. The DSC procedure for the binder-only tests was conducted at 25 °C (since the colder temperatures limit the extent of the photopolymerization reaction) and included a baseline step of 1 min, followed by UV irradiation at 20 W/m^2^ for 5 min. After that, this last step was repeated once more with the same UV dosage to deconvolute the heating effects due to the mercury lamp from the heat released during photopolymerization (similar to step C in Figure 3). The curve of heat flow versus time of this last procedure step was then subtracted from the heat flow of the first UV irradiation step to complete the deconvolution of heating effects. The Δ*H*_binder_ was then calculated from the entire exposure time
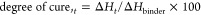
1Assessment of the crystallization of the binder components was also carried out on Mettler Toledo 3+ DSC. Each binder formulation as well as the individual monomers were loaded without particles or photoinitiator into the same 40 μL aluminum pans and twice heated and twice cooled between 25 and −70 °C at 10 °C/min to assess the crystallization behavior of the monomers used in the ink formulations.

### 2.8 TGA

Thermogravimetric analysis (TGA) was performed on a Mettler Toledo TGA2 STAR analyzer. Small sections (15−25 mg) of each control and TC prints for the three formulations were extracted for TGA at the conclusion of thermal cycling. The sampling of each small section was random in all cases. These TGA samples were loaded into 70 μL alumina crucibles and covered with a lid pierced with a 50 μm hole. The start temperature was 25 °C and was ramped to 500 °C at 10 °C/min. The atmosphere was N_2_ gas flowing at 200 mL/min to act as a purge gas.

## 3 Results and Discussion

**Figure 4:**
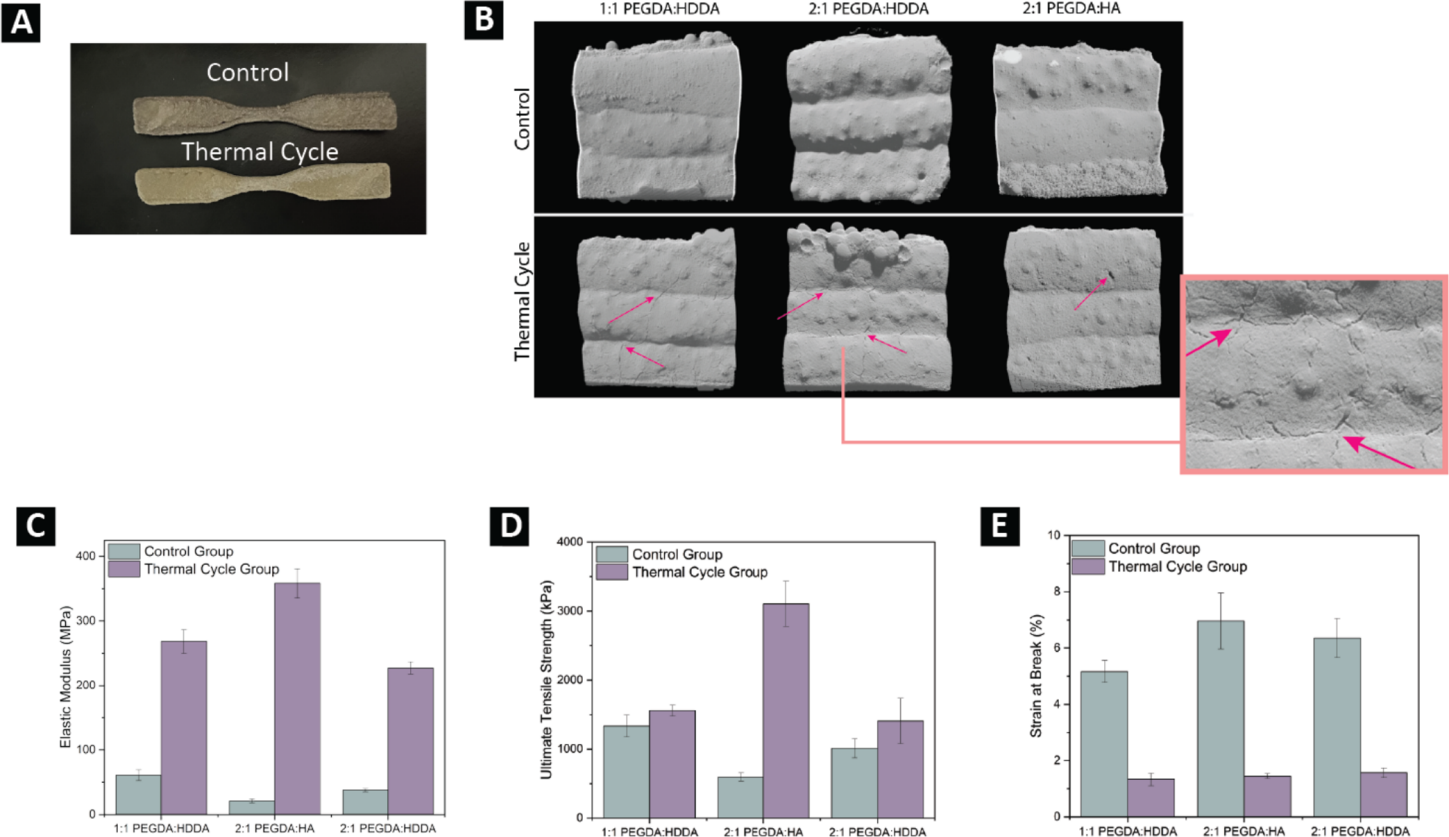
(A) Printed dogbones showing the color changes from the control group (top) to the yellowing on the TC (bottom) prints. (B) Close look at the printed dogbones imaged here with the Scanco μ50 scanner at a resolution of 3 μm/voxel. Side views of the prints are shown, limiting the scan volume but increasing the scan resolution. Visible microfractures (indicated with the arrows) can be seen in the TC group for all three formulations. One representative scan is shown out of three total scans for each formulation and thermal treatment. (C–E) Mechanical properties recorded from tensile testing four dogbones for each formulation in the control group (teal) and in the TC group (purple). (C) Elastic tensile modulus, (D) ultimate tensile strength, and (E) strain at break. Error bars represent the standard error of the mean of the four samples tested for each formulation and thermal treatment.

Prints were successfully produced for 1:1 PEGDA/HDDA, 2:1 PEGDA/HDDA, and 2:1 PEGDA/HA. The 1:1 PEGDA/HA did not result in consistent extrusion behavior with frequent leakage of the binder that altered the rheological properties of the ink and resulted in nozzle clogging. As a result, this formulation was discarded from continuing analyses as not enough samples could be reliably printed. For each of the nine samples printed from the other three formulations, five samples were exposed to thermal cycling, while four remained as a control group. The effect of thermal weathering was seen through physical changes on the dogbones, depicted through Figure 4a–e.

As seen in Figure 4a, the most noticeable effect of thermal cycling was a color change observed for the TC samples, with the prints appearing lighter and more yellow. Taking a closer look at the surface of the prints, imaged via microCT and shown in Figure 4b, the thermal cycle samples also present more microfractures on the exposed surface of the prints compared to the smoother exterior surface of the control samples. More microfractures appear to be present for the 1:1 PEGDA/HDDA and 2:1 PEGDA/HDDA formulations, with only minor instances observed for the 2:1 PEGDA/HA formulations. When considering the tensile test results presented in Figure 4c−e, the differences between the control and TC samples also occur beyond visual observations. There are some statistically significant (*t* test) differences observed for all formulations, both between the control and thermal cycle group, where thermal cycling drastically increases the elastic modulus of all formulations, and the elastic modulus also changes significantly (*t* test) when comparing formulations within either the control or TC group. In the control group, the elastic modulus is initially higher for the 1:1 PEGDA/HDDA prints, followed in order by the 2:1 PEGDA/HDDA and 2:1 PEGDA/HA prints. After thermal cycling, this order changes with the highest elastic modulus appearing in the 2:1 PEGDA/HA prints, followed in order by 1:1 PEGDA/HDDA and 2:1 PEGDA/HDDA. The ultimate tensile strength (UTS) does not change with thermal cycling of the HDDA-containing prints (*P* > 0.05, *t* test), but the 2:1 PEGDA/HA prints do see a large increase in UTS (Figure 4d). The strain at break is reduced by more than half after thermal cycling for all formulations, but there are no statistically significant (*t* test) differences between the different formulations.

From qualitative observations presented in Figure 4a,b, both the concentration of microfractures on the exposed surface of the prints and the color change after thermal cycling hint at erosion occurring due to weathering.^28^ However, with this kind of physical degradation, the stiffening observed in the tensile results is unexpected. One hypothesis is that the heating cycle could provide enough molecular mobility to the binder to rearrange in more favorable configurations, which could have resulted in an improvement in the tensile properties. On the other hand, the increase in stiffness and brittleness is very substantial to be only attributed to molecular rearrangement. When paired with the yellowing of the samples, the change in tensile properties suggests that chemical degradation may also be at play.

Since the inks are only composed of the crosslinked polymer and particles, and the solid particles would require more elevated temperatures to exhibit permanent chemical or microstructural changes, we expect chemical degradation in the cross-linked binder to be dominant. The combination of the oxidative environment and long heating is known to cause changes in the covalent bonds in polymers. Contrary to the oxidative environment employed here, the Moon lacks an atmosphere, and therefore, the increased material degradation due to the presence of oxygen in this study would not be encountered on the lunar surface. However, despite the lack of oxidative degradation, other aspects of the lunar environment, such as solar and cosmic radiation, may also cause chemical degradation within these materials. As we were unable to conduct the weeklong heating in vacuum, we elected to utilize an oxidative atmosphere to study how more significant degradation would affect the material properties of the formulations studied here, which could be caused by radiation on the lunar surface.

**Figure 5:**
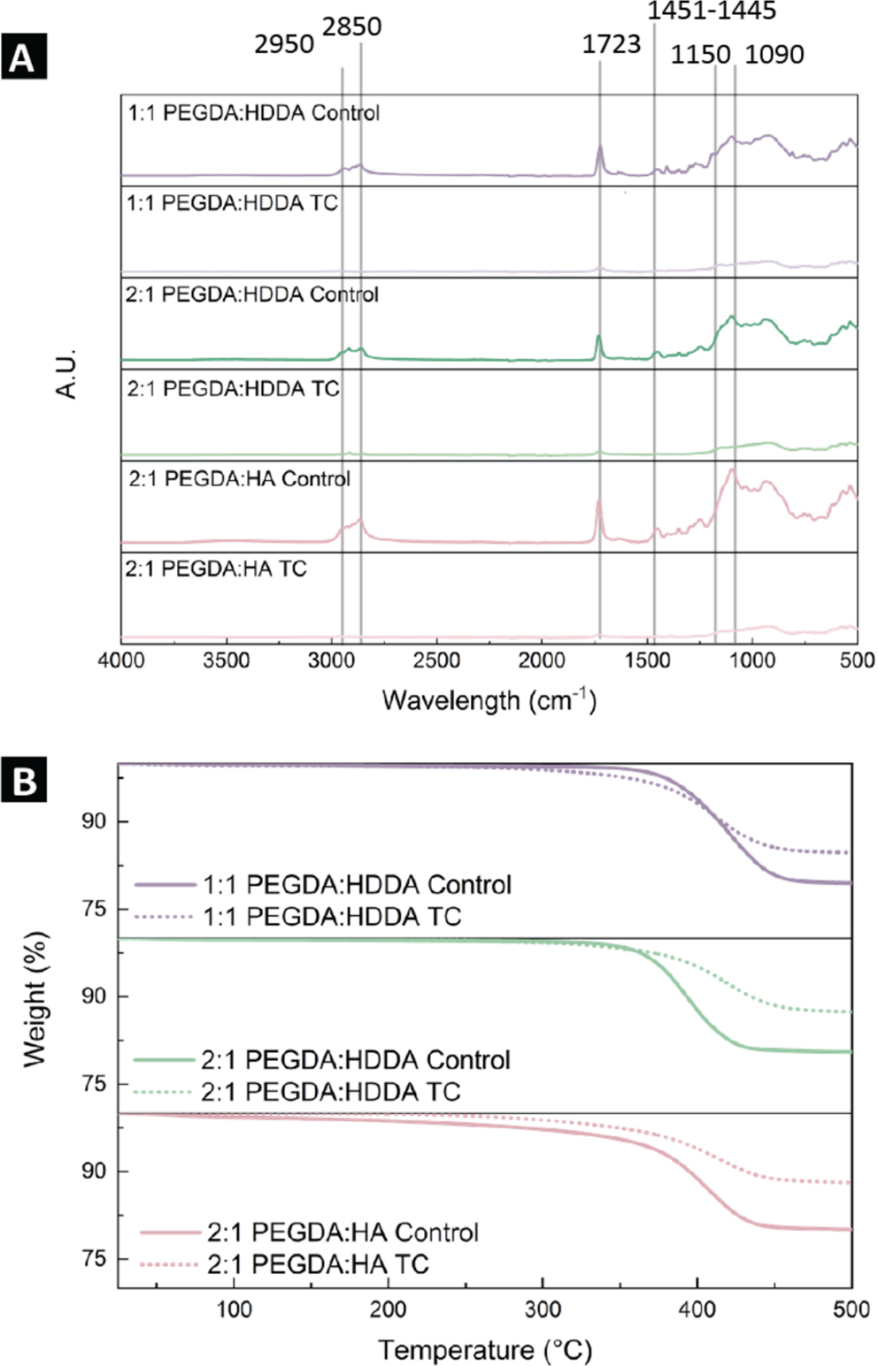
(A) FTIR spectra of the three formulations at both thermal treatments. Spectra are not normalized due to the changing intensity of the 1723 cm^−1^ peak. An average of three spectra for each formulation and thermal treatment are plotted in each case. (B) TGA curves averaged for three samples for each formulation and thermal treatment with heating from 25 to 500 °C. The plateaus after 400 °C indicate complete burnout of the binder and only particles remaining.

Considering the different increases in stiffness for the three binder formulations, it is important to assess how this degradation occurs with the different monomers and monomer ratios. Previous works on thermal cycling of cross-linked polymers have used peak intensity changes measured through FTIR to identify the bonds where functional groups were altered, while TGA can give insights into the overall binder amount within a composite sample.^44−49^ To this end, we present the spectra for all formulations with both thermal treatments in Figure 5a, supplemented by thermogravimetric data in Figure 5b. Figure 5a could not be normalized since the C=O peak at 1723 cm^−1^, traditionally used for FTIR normalization in evaluations of acrylate polymerization, is a potential site for bond cleavage during thermal degradation in an oxidative environment.^44,45^

For all three binder formulations, several peaks decrease in intensity or disappear after thermal cycling. Notably, the doublet near 2950−2850 cm^−1^ representing C−H stretching decreases, the peak at 1723 cm^−1^ representing the C=O bond decreases, the one at 1451−1445 cm^−1^ for C−O−O vibration stretching disappears, and the 1090 cm^−1^ convoluted peak for Si−O and C−O stretching^50^ in the control group shifts to 1150 cm^−1^ in the TC samples. Differences in the binder content of the three formulations are also observed in the TGA results in Figure 5b, where the amount of binder decreases by 24.8% for 1:1 PEGDA/HDDA, 36% for 2:1 PEGDA/HDDA, and 39.7% for 2:1 PEGDA/HA between the control and TC samples. The hypothesis of chemical degradation of the binder appears to be supported by both the FTIR spectra and the TGA curves. Degradation of the binder is supported with the decrease in C−H stretching bands at 2950−2850 cm^−1^, as well as the peak loss at 1347 cm^−1^ for the TC samples.^47,48^ These, supplemented by the yellowing observed in the TC dogbones, seem to indicate the formation of carbonyl compounds as a result of oxidative degradation during thermal cycling.^44,45,51^ However, the peak intensity decrease at 1723 cm^−1^, indicative of the C=O bond, also suggests loss of those carbonyl compounds in the TC samples.^47,48^ This point is supported by the lower amount of binder in the TC samples revealed by the TGA curves shown in Figure 5b, as well as the decrease in sample mass observed upon isothermal heating at 127 °C for 1 h (SI Figure 3) and can indicate that the decrease in binder mass in the prints after thermal cycling could be a result of outgassing.

**Figure 6:**
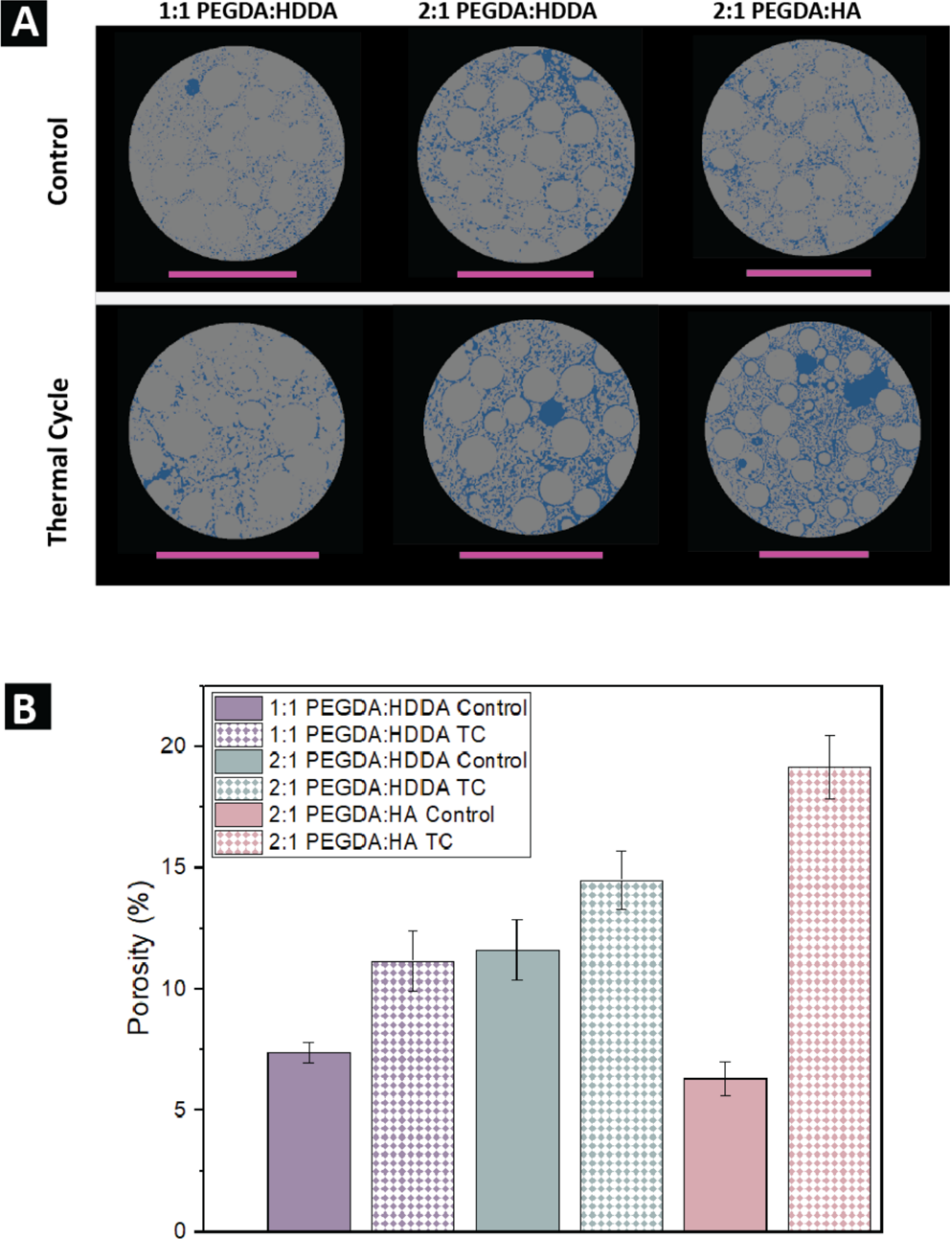
(A) Thresholded images of a single slice of each formulation and thermal treatment. One slice shown out of three total scans thresholded for each formulation and thermal treatment. Particles and binder are represented in gray, and pores/voids are represented in blue. Scale bars shown in pink all indicate 1 mm. (B) Measured porosity values after thresholding the three formulations at both thermal treatments with Otsu’s method. Error bars represent the standard error of the mean of three samples’ porosities.

The loss of carbonyl compounds is likely due to physical degradation happening concurrently with chemical degradation. Due to a difference in coefficients of thermal expansion (CTE) between the particles and the binder, the porosity of the prints is expected to increase with thermal cycling. The mechanism behind this is deadhesion between the binder and the particles, as the binder is more prone to expansion and contraction during heating and cooling cycles, respectively.^24,30,32^ However, both the high content of solid particles and the differences in binder formulations make it challenging to determine to what extent the porosity might increase with thermal cycling. Therefore, the porosity of segments of printed dogbones was assessed via Otsu’s method of thresholding and is shown qualitatively and quantitatively in Figure 6a,b. As observed for all three formulations, porosity increases with thermal cycling. In Figure 6a, the presence of voids along the glass microsphere/binder interface is notable for the TC samples, with the highest porosity reported for the 2:1 PEGDA/HA ink. The results in Figure 6b show that porosity increases by 51.2, 24.7, and 203.8% for 1:1 PEGDA/HDDA, 2:1 PEGDA/HDDA, and 2:1 PEGDA/HA, respectively. When only the control samples are compared, the highest porosity is observed in the 2:1 PEGDA/HDDA sample, followed by both the 1:1 PEGDA/HDDA and 2:1 PEGDA/HA ink formulations. For a similar comparison among thermal cycle samples, the order of decreasing porosity changes, with 2:1 PEGDA/HA having the highest porosity, followed by 2:1 PEGDA/HDDA and 1:1 PEGDA/HDDA.

The porosity data presented in Figure 6a,b show that despite the increase in stiffness of the prints after thermal cycling, the printed dogbones still become more porous. The increase in porosity for the different formulations is also tied to the CTEs measured for the three binders, with 1:1 PEGDA/HDDA featuring both the lowest CTE, at 189.3 ppm/°C, and the smallest increase in porosity with thermal cycling. The CTEs for 2:1 PEGDA/HDDA and 2:1 PEGDA/HA are higher, at 266.3 and 243.3 ppm/°C, respectively, and create a larger mismatch with the CTE of the solids (i.e., the CTE for glass particles is 8.7 ppm/°C^52^), which results in higher porosity after thermal cycling. With regard to the chemical degradation noted in Figures 4 and 5, it is likely that both expansion and contraction of the binder and deadhesion at the particle–binder interface facilitate the production of pathways of pores across the print volume through which carbonyl degradation products can escape. This would justify the observed decrease in carbonyl peak intensity and lower binder amount for the TC samples in Figure 5a,b. Therefore, chemical and physical degradation are likely taking place at the same time during thermal cycling.

**Figure 7:**
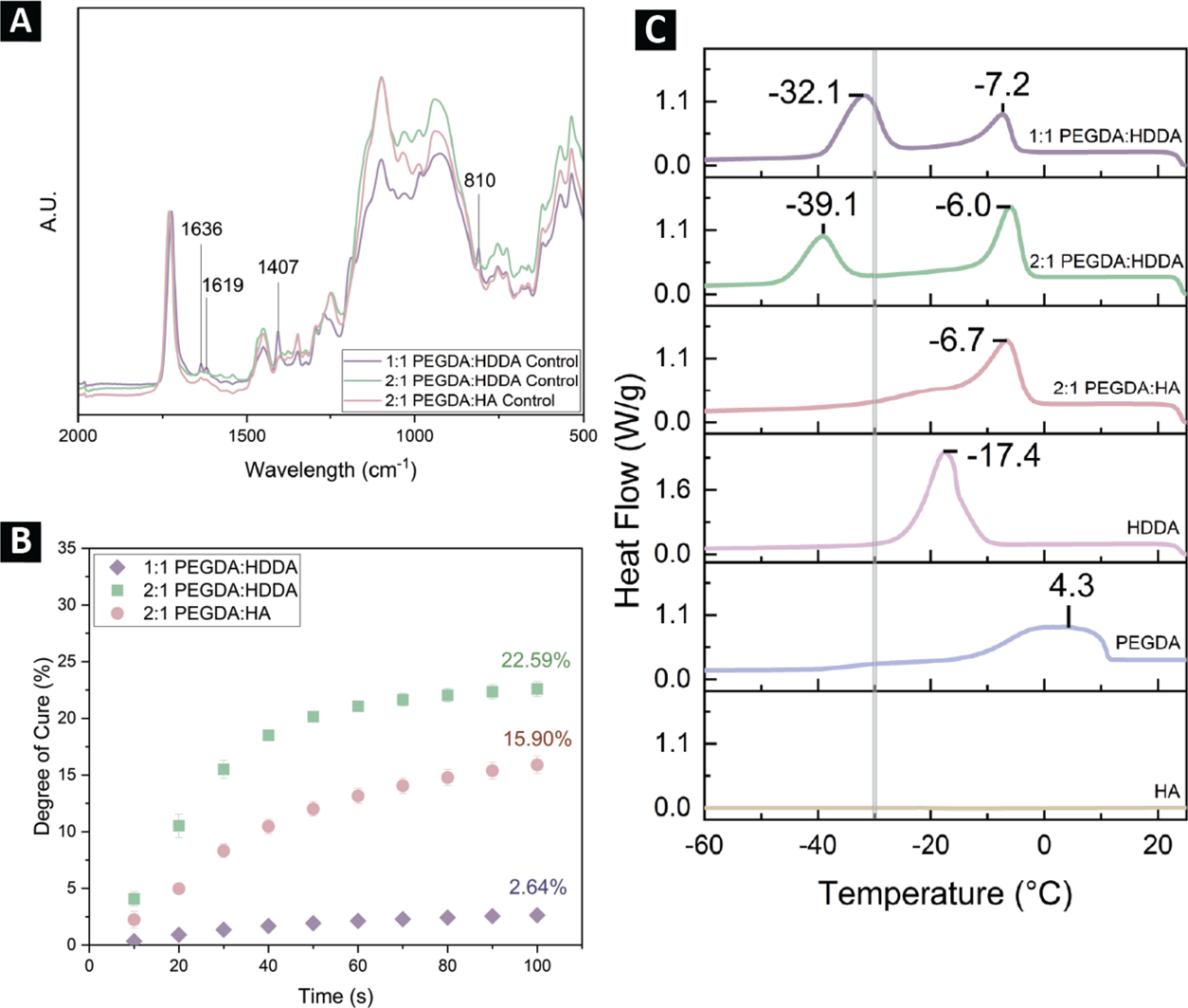
(A) Spectra of the control group and each of the three formulations normalized at the 1723 cm^−1^ peak. Key peaks related to the incomplete conversion of vinyl bonds are denoted. The C=C peak is indicated at 810 cm^−1^ and is visible for the 1:1 PEGDA/HDDA sample. The 1636 and 1619 cm^−1^ peaks representing C=C bonds are also visible in the 1:1 PEGDA/HDDA sample, and the peak at 1407 cm^−1^ indicates a methyl group likely present on the unreacted BAPO photoinitiator, also in the 1:1 PEGDA/HDDA ink. Each plotted spectrum is the average of three repeat scans. (B) Degrees of cure for the four printing formulations at −30 °C recorded from the photo-DSC data and converted with eq 1. Error bars represent the standard error reported for the mean of the three repeat samples. (C) DSC cooling curves shown for each of the three binder formulations and the individual binder components. The temperature at each of the crystallization peaks is indicated, and the printing temperature of −30 °C is highlighted with a gray vertical line.

Thus far, we have shown that all three binder formulations demonstrate microstructural changes as a result of thermal cycling. However, each formulation featured different degrees of increase in stiffness and porosity (shown in SI Figure 4), likely because the monomers and ratios were selected so that the final cured products would feature varying levels of cross-linking. This may indicate that the physical and chemical degradation mechanisms discussed are dependent on the binder composition and processing. Therefore, we investigated in more detail the formation of the cross-linked structure for each binder formulation during printing at −30 °C. In Figure 7a, the FTIR spectra of the control samples are presented with normalization. Then, Figure 7b demonstrates more insights into the polymerization kinetics at −30 °C through the degree of cure measured via photo-DSC. Finally, in Figure 7c, the thermal behavior of the individual monomers and uncured binder formulations covering a wide range of temperatures is shown.

As illustrated in Figure 7a, the spectra for the 1:1 PEGDA/HDDA prints feature sharp peaks for C=C bonds at 810, 1636, and 1619 cm^−1^ that are absent in the 2:1 PEGDA/HDDA and 2:1 PEGDA/HA inks. Likewise, there is another distinct peak at 1407 cm^−1^ representing −CH_3_ that appears only in the 1:1 PEGDA/HDDA spectra. These clearly indicate the remaining presence of uncured ink and leftover photoinitiator molecules in the 1:1 PEGDA/HDDA ink. Looking at this same formulation in the TC spectra in Figure 5a (larger graphs are included in SI Figure 2) indicates that unreacted monomers have been converted after thermal cycling. The degree of cure presented in Figure 7b further supports the presence of unreacted vinyl bonds in the 1:1 PEGDA/HDDA ink. As can be observed, the degree of cure is low for all formulations, not surpassing 30% even after 100 s of UV exposure at 20 W/m^2^. In the case of the 1:1 PEGDA/HDDA mixture, very low cure degrees are achieved at this temperature. For the 2:1 PEGDA/monomer mixtures, the degree of cure for the HDDA monomer increases more quickly than the HA monomer in the first 60 s of exposure and reaches a higher degree of cure even after plateauing. The low degrees of cure for these inks are not unexpected, considering the large number of opaque particles incorporated in the formulations. In our past work, printing in ambient temperatures with similar inks did not produce cure degrees above 50%.^38^ These results were nonetheless surprising considering that the 1:1 PEGDA/HDDA ink contains the highest number of reactive vinyl groups, compared to 2:1 PEGDA/HDDA and 2:1 PEGDA/HA, and was hypothesized to be the most cross-linked of the three binder formulations.

The differences in curing between monomer systems tie in with the observed change in yielding behavior of the uncured inks between 25 and −20 °C, as shown in SI Figure 5. A higher increase in yield point as the temperature drops corresponds to lower degrees of cure at −30 °C. The increase in yield points observed in SI Figure 5 and the cure degrees shown in Figure 7b are likely results of the density of the binders (mass average densities: 1.068 1.103, and 1.088 g/mL for 1:1 PEGDA/HDDA, 2:1 PEGDA/HDDA, and 2:1 PEGDA/HA, respectively), where less dense binder formulations facilitate the growth of crystals by enabling the diffusion of monomers. Binder density can therefore be a key parameter in formulations printed in extreme cold, as a lower-density promoting monomer crystallization limits the extent of cure of the printed parts. By comparison of the 2:1 PEGDA/monomer formulations, the higher cure degrees achieved with the HDDA monomer, compared to the HA monomer, support the practice of using monomers with higher functionality to promote the extent of cure. This suggests that common formulation strategies to improve curing in suspensions of opaque particles are not as straightforward when attempting to promote curing at subzero temperatures.

In our prior work, we demonstrated the impact of subzero printing temperatures on slowing down curing reaction kinetics due to crystallization of the PEGDA monomer. Therefore, we assessed the potential for crystallization of both HDDA and HA monomers as well as for the monomer mixtures. Shown in Figure 7c, endothermic crystallization peaks are present for both PEGDA and HDDA monomers and also appear in mixtures of the two, while the HA monomer does not crystallize. For the PEGDA/HDDA mixtures, two crystallization peaks are apparent. When the ratio is 1:1 PEGDA/HDDA, the onset of the first peak is −4.9 °C (attributed to PEGDA crystallization) and the onset of the second peak is −27.3 °C (attributed to HDDA crystallization). In the 2:1 PEGDA/HDDA ratio, the onsets of the first and second peak are −3.1 °C (attributed to PEGDA crystallization) and −34.2 °C (attributed to HDDA crystallization), respectively. Compared to pure PEGDA, the mixtures feature a shift in PEGDA crystallization to lower temperatures due to amorphous monomer inhibiting the growth of PEGDA crystals (HDDA is still considered amorphous at the PEGDA crystallization temperatures). In the 2:1 PEGDA/HA formulation, a single peak is observed at −6.7 °C (also attributed to PEGDA crystallization) with a shoulder trailing to lower temperatures. When deconvoluted, the secondary peak appears at −20.13 °C. This shoulder is likely due to a broader range of crystal sizes forming out of the PEGDA chains when the HA monomer is included. Since HA does not crystallize, it can act as an inhibitor to PEGDA crystallization, shifting the onset crystallization temperature to lower temperatures and impacting the size of crystals forming. While the first peak in the PEGDA-HDDA mixtures, attributed to PEGDA crystallization, occurs at warmer temperatures than −30 °C, we have previously shown how the heat released during polymerization is sufficient to melt PEGDA crystalline regions and allow for curing at −30 °C.^33^ However, this heat can only be initially generated with polymerization of a noncrystallizing monomer, such as HA. In the case of the 1:1 PEGDA/HDDA monomer ink, the onset of crystallization before −30 °C restricts both monomers from having sufficient mobility for polymerization. When the ratio of PEGDA to HDDA is changed to 2:1 instead, HDDA monomers retain mobility and can polymerize at −30 °C, since the onset of HDDA crystallization (the second crystallization peak) occurs below the printing temperature. This is likely why the degrees of cure in Figure 7a are the lowest for the 1:1 PEGDA/HDDA ink and why vinyl bonds can be detected in the FTIR spectra in Figure 7b, whereas the 2:1 PEGDA/HDDA ink achieves higher levels of curing and vinyl bond conversion. These results highlight the importance of optimizing the monomer ratio to produce binders whose thermal behavior does not impair printing solidification.

The effect of binder crystallization and the resulting low degrees of cure are carried through to the mechanical properties and degradation of the samples. Since the FTIR spectra of the TC samples do not identify the remaining vinyl bonds, it is likely that the heating cycle of thermal cycling induces these monomers to react. We hypothesize that the weeklong heating melts crystalline regions that can then free trapped radicals or vinyl groups.^35^ Since cure shrinkage is common with acrylate monomers,^53^ continued polymerization during thermal cycling could have contributed (in addition to degradation) to the increase in porosity observed in Figure 6b, particularly for the 1:1 PEGDA/HDDA ink. Likewise, the mismatch between the higher elastic moduli in the TC formulations despite the low degrees of cure after printing can indicate that residual curing is contributing to stiffening the prints. This can justify the 1351−1347 cm^−1^ stretch disappearing in the spectra of the TC samples,^54^ as seen in Figure 5a, and indicates that oxidative chemical degradation may not be the sole mechanism at play behind increasing cross-linking. Instead, it is possible that residual curing of vinyl bonds and chemical degradation are competing mechanisms, particularly when comparing the mechanical properties of 1:1 PEGDA/HDDA and 2:1 PEGDA/HA formulations. Under ambient conditions, the extent of cure and cross-linking density of 1:1 PEGDA/HDDA would be higher than 2:1 PEGDA/HA, due to more reactive vinyl groups. However, with Figures 5a and 7a showing that curing continues during thermal cycling, it is possible that 1:1 PEGDA/HDDA still achieves a higher cross-linking density than 2:1 PEGDA/HA despite crystallization. Since higher cross-linking densities typically give materials better thermal stability,^34,35^ this could support Figure 4c, indicating less chemical degradation in 1:1 PEGDA/HDDA since it has a lower increase in elastic modulus with thermal cycling than 2:1 PEGDA/HA. Likewise, this could also support more degradation in the 2:1 PEGDA/HA ink, since in addition to having the highest increase in elastic modulus, it is the only ink that also has a higher UTS after thermal cycling. Therefore, these results demonstrate that monomer crystallization at −30 °C and residual curing likely affect the progression and extent of thermal degradation mechanisms in polymer–regolith composites.

## 4 Conclusions

The potential for 3D-printed components composed of ISRU materials, such as regolith particles, to be routinely used in the thermal extremes of the lunar surface depends on the interplay between the ink formulation and processing conditions. In this work, we created and printed several high solid inks from candidate formulations composed of lunar regolith simulant LHS-1 and glass microspheres, both of which were suspended in UV-curable binders. We replicated partial lunar processing conditions by carrying out the printing process at −30 °C, and we evaluated the resulting prints’ performance after exposure to replicated lunar day/night temperatures (127 and −190 °C, respectively). The binders in the ink were intentionally varied to create formulations with different reactivities to UV curing and to produce prints with different cross-linking densities so as to assess the impact on weatherability of printed parts. Most importantly, we showed that the UV-cured binder is prone to both physical and chemical degradation and that monomer crystallization may play a part in limiting the extent of both degradation mechanisms.

The thermal cycling treatment conducted here increased the stiffness of all inks, regardless of formulation, indicating chemical changes within the ink. The increases in porosity and surface erosion observed in all samples also support physical deterioration. We demonstrated that these phenomena occur with the formation and probable outgassing of carbonyl compounds, while residual trapped vinyl bonds cure and increase cross-linking during the heating cycles of thermal cycling. The extent to which both occur appears to depend on the interactions between PEGDA and the HDDA monomer and their ratios in a binder mixture. When utilized in a 1:1 ratio, we showed that crystallization of both monomers restricts the initial UV cure polymerization, which is resumed during thermal cycling as a potential competing mechanism for chemical degradation. In contrast, in a 2:1 ratio, only one monomer crystallizes; therefore, initial curing is higher and residual curing during thermal cycling is likely dominated by chemical degradation mechanisms. This highlights the importance of optimizing not just the cross-linking density and reactivity of the monomers but also their crystallization potential when printing at extremely low temperatures, as differences in crystallization propagate through to differences in the thermal stability of the solidified materials. Overall, with these results, we demonstrated how the thermal behavior of uncured ink carries from print solidification to the degradation behavior of printed parts. Although this work only investigates thermal weathering, we expect that it will contribute to future ISRU efforts by providing insights into the usage life of regolith–polymer composite feedstocks for in-space manufacturing.

## Supporting Information Available

Additional information includes microCT renderings of entire dogbones; measured coefficients of thermal expansion of the binders; and unnormalized FTIR spectra of TC samples (PDF)
