# Supplementary material for: Thermal Weathering of 3D-Printed Lunar Regolith Simulant Composites
Source: ACS Appl Eng Mater. 2024 Jul 27;2(8):2016–26. doi: 10.1021/acsaenm.4c00158 (PMC11348411; doi:10.1021/acsaenm.4c00158)
Supplement: Supplementary file 1 — em4c00158_si_001.pdf [file em4c00158_si_001.pdf]

Supporting Information

Thermal Weathering of 3D Printed Lunar Regolith Simulant Composites

Alexandra Marnot[1], Jami Miliken[2], Jaehyun Cho[1], Zihao Lin[3], Chingping Wong[3], Jennifer M. Jones[4], Curtis Hill[5], Blair Brettmann[1,3*]

[1]School of Chemical and Biomolecular Engineering, Georgia Tech, Atlanta GA 30332

[2]Daniel Guggenheim School of Aerospace Engineering, Georgia Tech, Atlanta GA 30332

[3]School of Materials Science and Engineering, Georgia Tech, Atlanta GA 30332

[4]NASA Marshall Space Flight Center, Huntsville AL 35806

[5]NASA Marshall Space Flight Center, Jacobs Space Exploration Group, Huntsville AL 35806

* corresponding author, blair.brettmann@chbe.gatech.edu

A. microCT renderings of the entire dogbones

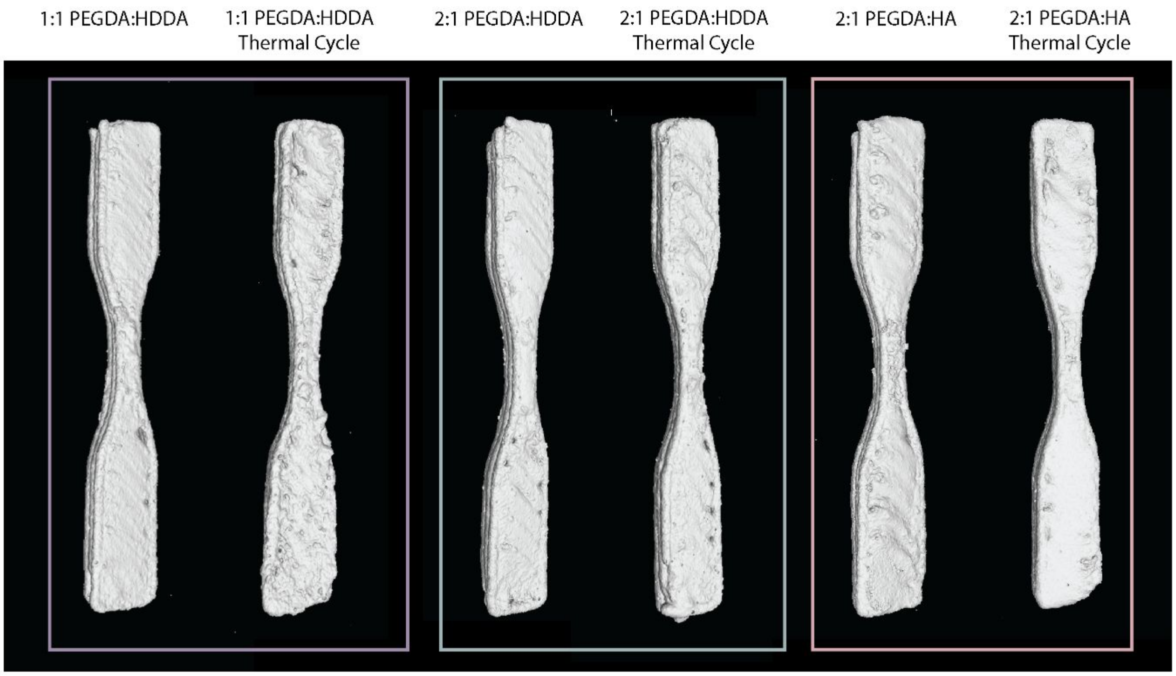

SI Figure 1: Large scale micro-CT scans on 1 representative dogbone from each formulation and thermal treatment. The samples are represented upright, at a slight angle, with the top surface facing the front of the page.

B. CTE values

SI Table 1: Coefficients of thermal expansion measured for the three binder formulations collected from 40°C to 150°C at 10°C/min on a TMA Q400 (TA Instruments) with expansion probe accessory. The average of three repeat measurements is presented, along with the standard error.

	**1:1 PEGDA:HDDA**	**2:1 PEGDA:HDDA**	**2:1 PEGDA:HA**
**Coefficient of Thermal Expansion (ppm/°C)**	189.3 ± 8.7	266.3 ± 13.0	243.3 ± 6.0

C. Unnormalized FTIR spectra of the TC samples

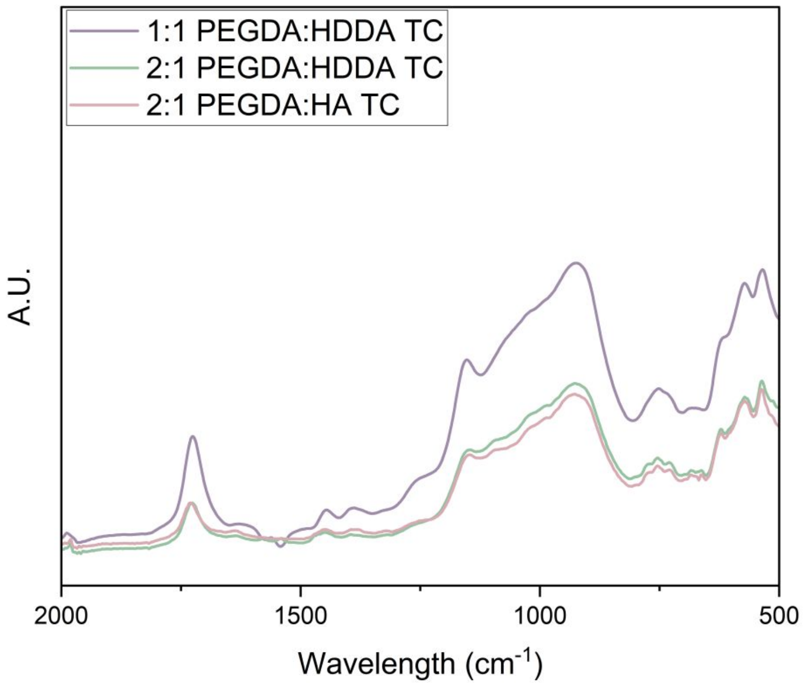

SI Figure 2: FTIR spectra (unnormalized) of the printed samples that underwent thermal cycling for the three formulations studied. Larger view for comparison with Figure 6a in the main text. Each spectrum is the average of three scans

.

D. Isothermal TGA

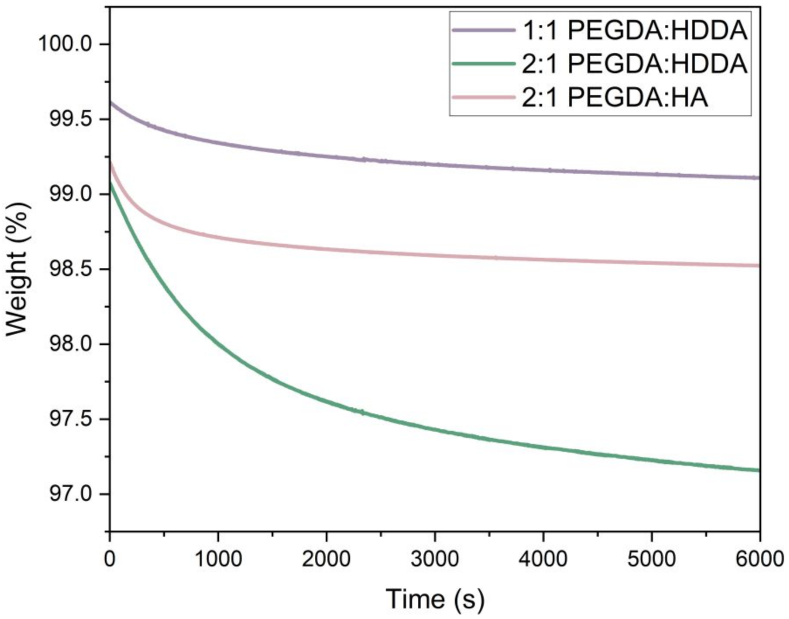

SI Figure 3: Decrease in sample mass with an 1h hold at 127°C. It is expected that the decrease in mass is a result of chemical degradation occurring in the binder and the outgassing of carbonyl compounds. However, the decrease in mass shown here is still likely less than occurred during the heating periods of thermal cycling, as these TGA experiments were conducted in N_2 whereas the thermal cycling heating periods were conducted in air.

E. Comparison of the increase in stiffness and increase in porosity

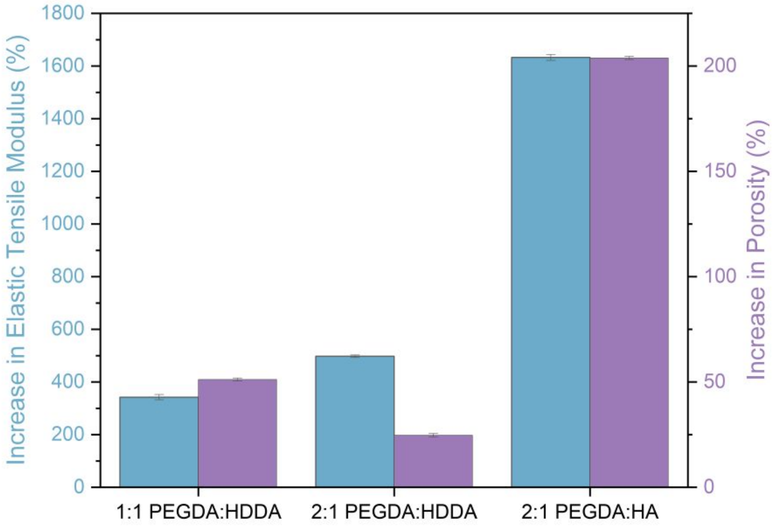

SI Figure 4: Comparison of the increase in stiffness (measured through the elastic tensile modulus) and the increase in porosity of the three formulations studied between the control group and the TC group. The error bars are the propagated standard error of the mean values of elastic modulus and porosity that were computed for each of the formulation in both the control group and the TC group.

F. Yield Point Rheology

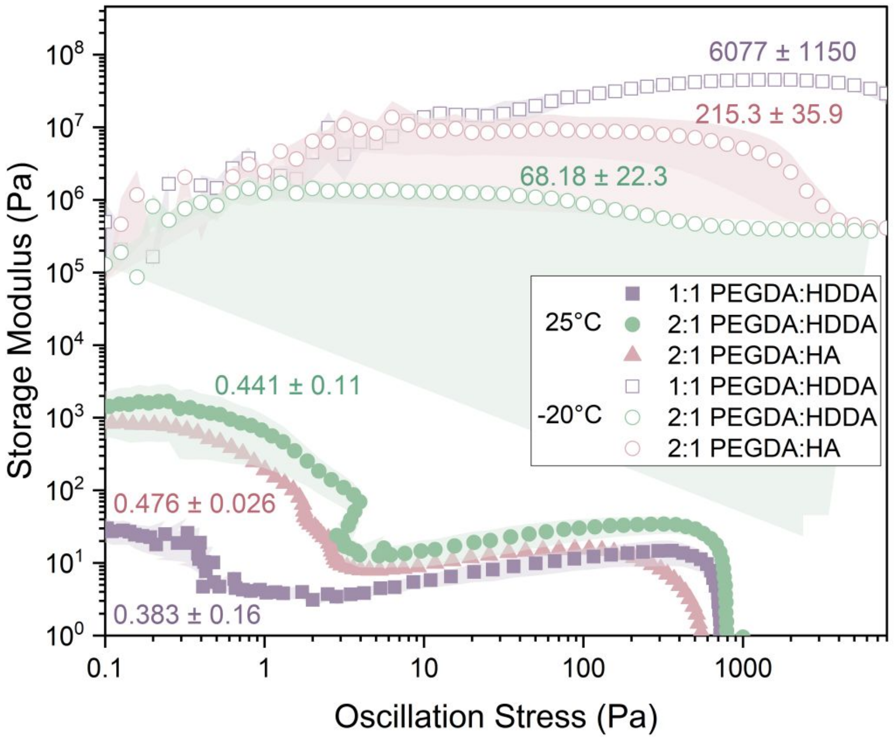

SI Figure 5: Yielding curve and yield points of the different inks with increasing oscillation stress at 25°C (filled symbols) and -20°C (hollow symbol), which was the lower limit of our chiller, although we aimed for -30°C.
